# Supporting service quality improvement for pharmacy-based alcohol screening and brief advice services

**DOI:** 10.1186/1940-0640-8-S1-A44

**Published:** 2013-09-04

**Authors:** Adam J Mackridge, Elizabeth C Stokes, Janet Krska

**Affiliations:** 1 School of Pharmacy & Biomolecular Sciences, Liverpool John Moores University, Liverpool, Merseyside, UK; 2 Medway School of Pharmacy, The Universities of Greenwich and Kent at Medway, Chatham, Kent, UK

## Introduction

Pharmacy-based alcohol SBI services have been commissioned across England but evidence on quality is limited. This study developed methods to assess quality and support development. The study was funded by Liverpool PCT using an unrestricted educational grant from Lundbeck Limited.

## Methods

Five pharmacies providing alcohol SBI services in three areas were purposively selected and data collected on service delivery and quality, via three methods: 1) Trained market researchers (MRs) covertly completed a structured checklist for each pharmacy on environment and service promotion, and requested a hangover treatment, recording experiences and outcomes of services offered. 2) MRs observed interactions between pharmacy staff and customers. Field notes described pharmacy activity and customer interactions. 3) In two pharmacies, IBA service consultations were recorded and service users' views gained by interview 2 weeks later. Individual feedback reports were discussed with pharmacy staff at each pharmacy in a group interview. Feedback comprised simulated experience of service provision, opportunities to offer services, and, in two pharmacies, service user experience.

## Results

Promotion and pharmacy environment were variable in quality from the simulated user perspective and not all MR requests for hangover remedies resulted in an offer of the SBI service. Useful approaches were identified at different pharmacies, e.g. description of the service as an alcohol ‘quiz’. Medicines counter assistants provided 63% of initial interactions with customers, 76% of whom had presented a prescription for dispensing. Quality of questioning was variable, but the views of the 16 service users interviewed were positive. Pharmacy staff welcomed the individualised feedback and engaged in constructive discussion around facilitators and barriers to service provision as well as scope to improve quality.

## Discussion

Pharmacy staff were willing to participate in all stages of this project, despite some intensive observation, and feedback was received positively, with positive engagement in discussion on service improvement.

